# Central Pulse Wave Velocity and Augmentation Index Are Repeatable and Reproducible Measures of Arterial Function

**DOI:** 10.1002/hsr2.70155

**Published:** 2024-10-28

**Authors:** Sophie L. Russell, Mushidur Rahman, Charles J. Steward, Amy E. Harwood, Gordon McGregor, Prithwish Banerjee, Nduka C. Okwose, Djordje G. Jakovljevic

**Affiliations:** ^1^ Research Centre for Health and Life Sciences (CHLS) Coventry University Coventry UK; ^2^ Research Centre for Physical Activity, Sport and Exercise Science (CPASES) Coventry University Coventry UK; ^3^ Department of Sport and Exercise Sciences, Institute of Sport Manchester Metropolitan University Manchester UK; ^4^ Research Centre for Healthcare and Communities Coventry University Coventry UK; ^5^ Department of Cardiopulmonary Rehabilitation, Centre for Exercise and Health University Hospitals Coventry and Warwickshire NHS Trust Coventry UK; ^6^ Warwick Clinical Trails Unit, Warwick Medical School University of Warwick Coventry UK

**Keywords:** arterial function, augmentation index, pulse wave velocity, reliability

## Abstract

**Background and Aims:**

Arterial function (specifically arterial stiffness) is an important cardiovascular risk factor. Pulse wave velocity (PWV) and augmentation index (Alx) are established indicators of arterial function. The present study aimed to evaluate the repeatability and reproducibility of PWV and Alx in healthy individuals.

**Methods:**

Forty healthy participants (age 33 ± 11 years, 17 females) underwent resting supine PWV and Alx assessments. Measurements were made in triplicate and repeated 1 week apart. Alx was measured by brachial occlusion and PWV was measured from the carotid artery to the femoral artery via the tonometer‐oscillometric method. Repeatability and reproducibility were assessed using the intraclass correlation coefficient (ICC). Interoperator reproducibility was performed on 10 participants.

**Results:**

The average values for week‐to‐week visits for PWV and Alx were 6.20 ± 0.91 versus 6.13 ± 0.91 ms^−1^ and 14.0 ± 11.8 versus 16.3 ± 12.2% respectively. For same‐day measurements, both PWV and Alx showed excellent repeatability (PWV: ICC = 0.96, 95% CI: 0.92–0.98, *p* < 0.01; Alx: ICC = 0.90, 95% CI: 0.84–0.94, *p* < 0.01) and interoperator reproducibility (PWV: ICC = 0.98, 95% CI: 0.93–1.00, *p* < 0.01; Alx: ICC = 0.93, 95% CI: 0.69–0.98, *p* < 0.01). Measurements were repeated 1 week apart and showed good reproducibility (PWV: ICC = 0.77, 95% CI: 0.61–0.87, *p* ≤ 0.01; Alx: ICC = 0.73, 95% CI: 0.73–0.86, *p* < 0.01).

**Conclusion:**

PWV and Alx demonstrate excellent repeatability and good reproducibility. Considering these variables are noninvasive and easy‐to‐measure, arterial function assessment may have a role in routine clinical practice to facilitate risk stratification in cardiovascular diseases.

## Summary

- PWV and Alx are repeatable and reproducible measures of arterial function
- PWV is more repeatable and reproducible than Alx but requires training before operation

## 1 Introduction

Arterial stiffness is a measure of vascular function and can provide additional useful information on cardiovascular risk not obtained with standard blood pressure measurement [1]. Arterial stiffening occurs as a natural consequence of aging and may be a precursor to disease [2]. Stiffening is accelerated by the build‐up of fatty streaks within the endothelium of blood vessels, particularly large arteries, and is a major factor responsible for cardiovascular disease in older adults [3]. Traditional cardiovascular risk factors such as smoking are known to accelerate arterial stiffening [4], while engagement in moderate‐to‐vigorous physical activity can limit its progression [5].

Arterial stiffness is independently associated with COVID‐19 infection [6], and can predict cardiovascular events in both those with coronary artery disease [7] and diabetes [8]. Increased arterial stiffness is common in people with heart failure with preserved ejection fraction, and is associated with disease progression (and mortality) in chronic kidney disease [9]. Wilkinson et al. indicated the potential advantage of measuring arterial stiffness in practice to identify at‐risk patients, but highlighted that this assessment should be used to risk stratify medium‐risk hypertensive patients rather than the high‐risk hypertensive patients, who are able to be stratified by standard blood pressure measurement alone [10]. However, clinical assessment of arterial stiffness is still not standard practice in the United Kingdom.

Pulse wave velocity (PWV) and pulse wave analysis (PWA) (with augmentation index [Alx] being an output of PWA) are commonly used to assess vascular function. PWV is a measure of the velocity of blood flow through an artery and is calculated as the ratio between the distance the pulse travels and the time it takes to travel down the artery [1]. In multivariate modeling, Mitchell et al. discovered that adding PWV into the Framingham risk prediction increased the cardiovascular disease risk predictive value by 0.7% [11]. The 2018 European Society of Hypertension guidelines suggest a value of carotid to femoral PWV of 10 ms^−1^ requires further investigation [12], with reference values for age groups also available [13]. Carotid‐femoral PWV, as measured via the probe‐based method, is the gold standard assessment of arterial stiffness [2]. However, obtaining PWV requires skilled users with specific training [14].

PWA is an alternative method of measuring vascular function, requiring only brachial occlusion, thus making it a considerably easier method than PWV. PWA provides values for both augmentation pressure (the reflected brachial pulse wave) and pulse pressure (difference between systolic and diastolic pressures) [14]. Alx is then calculated as the percentage of the ratio between augmentation pressure and pulse pressure. The common mechanism of increase in Alx is faster forward pulse propagation and a more rapid reflected wave [14]. In a meta‐analysis, Alx has been shown to predict clinical events independently of peripheral blood pressure [15] and as Alx increases linearly until age 50, this could be a more sensitive marker of arterial aging in younger adults [16].

Previous studies have shown that the Sphygmocor MM3 technology which utilizes applanation tonometry to assess stiffness, is reliable [17, 18]. However, there is limited research evaluating the functionality of the most recent modes of arterial stiffness assessment, which use tonometry as well as occlusion. Thus, the aim of the current study was to evaluate the reliability and repeatability of both PWV and Alx in adults utilizing the Sphygmocor XCEL (tonometry and occlusion) technology.

## 2 Methods

### 2.1 Study Design and Procedure

To ensure continuity of reporting in future research, the following study is reported in accordance with the Guidelines for Reporting Reliability and Agreement Studies (GRRAS) [19]. Healthy adult volunteers aged between 18 and 65 years were recruited for the study. Data were collected between April 2021 and January 2022. Healthy participants were defined as those without history of chronic or acute cardiovascular, respiratory, or neurological conditions. Participants were excluded if they used medication that was known to effect cardiovascular function, they were a current smoker, or their body mass index (BMI) was greater than 35 kg m^−2^. Ethical approval was obtained through the Coventry University Research Ethics Committee (project reference number: P109193). Participants were required to attend the clinical physiology laboratory on two occasions (1 week apart and at the same time of day) and provide written informed consent.

Participants were asked to lay in the supine position for at least 10 min in a temperature‐controlled room before taking arterial function measurements. The Sphygmocor Xcel unit (AtCor Medical, Naperville, Illinois, USA) was used to measure arterial function. Alx was obtained using a brachial cuff, which recorded a blood pressure and pulse pressure measurement from the left brachial artery (approximately midway between the shoulder and the elbow). Measurements were performed in triplicate, and all measures passed the internal quality control criteria [20]. PWV measurement used applanation tonometry over the carotid artery and a partly inflated cuff over the top of the thigh. Assessment required participant height, sex, date of birth, and pulse transit distance. Pulse transit distance was obtained by subtracting the distance from the carotid artery to the sternal notch and the cuff to the femoral pulse (predetermined at 200 mm to avoid need for invasive procedure) from the distance from the sternal notch to the top of the femoral cuff. When appropriate signals from both the cuff and tonometer were obtained, concurrent femoral and carotid pulse waves were captured for a period of 10 s. The internal quality control criteria were used to ensure quality of data [20].

Participants returned 1 week later, at the same time of day, to repeat all measures. A subset of 10 participants underwent inter‐operator reproducibility, with a second, trained operator obtaining triplicate values for Alx and PWV, during the same period that the participant was in the supine position.

### 2.2 Statistical Analysis

Data were analyzed using the SPSS statistical package version 26 (SPSS Inc., Chicargo, IL, USA). Data are expressed as mean ± standard deviation, unless otherwise indicated. Before statistical analysis, data were screened for univariate and multivariate outliers using standard Z distribution cut‐offs. Intraclass correlation coefficient (ICC) was performed to determine repeatability, interrater reproducibility, and week‐to‐week reproducibility. All outcomes were prespecified, but the study was powered for intratest repeatability.

Intratest repeatability was based on single measure, absolute agreement, two‐way mixed effect model. Interrater and week‐to‐week reproducibility was based on a mean rating (*k* = 3), single rater, absolute agreement, and two‐way mixed effect model. Values of < 0.5, 0.5–0.75, 0.75–0.9, and > 0.9 were considered poor, moderate, good and excellent reliability respectively [21].

To inform the sample size, a power calculation was performed. With a minimum acceptable ICC of 0.7, an expected ICC of 0.9, two repetitions per participant, an alpha of 0.05% and 95% power, 37 participants were required. Accounting for a 10% dropout rate, 42 participants would need to be recruited [22].

## 3 Results

A total of 40 individuals aged between 22 and 58 years old were recruited to the study. Due to dropout being less than expected, only 40 participants were recruited which meant that the powered target of 37 individuals was achieved. Demographic and anthropometric characteristics of the participants are presented in Table 1. Participants were classified as healthy with an average BMI of 25 kg/m² and blood pressure of 124/77 mmHg. Mean and standard deviations of PWV and AIx measures for intra‐, inter‐, and test‐retest variability time points are presented in Table 2.

**Table 1:** Demographic and physical characteristics of study participants.

Parameter	Mean ± SD	Range
Age	33.4 ± 11.0	22–58
Sex, male/female	23/17	
Height (cm)	174 ± 8.4	158–190
Weight (kg)	76.4 ± 13.3	49–114
BMI (kg/m^2^)	25 ± 3.70	18.3–37.4
Systolic blood pressure (mmHg)	124 ± 11.7	100–142
Diastolic blood pressure (mmHg)	76.8 ± 8.39	63–98

Abbreviations: BMI, body mass index; SD, standard deviation.

**Table 2:** Mean and standard deviation of augmentation index (Al x) and PWV.

		PWV (mean ± SD, ms^−1^)	Al x (mean ± SD, %)
Intratest repeatability	Repeat 1	6.17 ± 0.89 (*n* = 40)	14.2 ± 9.81 (*n* = 40)
	Repeat 2	6.12 ± 0.97 (*n* = 40)	14.6 ± 10.7 (*n* = 39)
	Repeat 3	6.05 ± 0.94 (*n* = 37)	13.9 ± 10.6 (*n* = 39)
Test−retest reproducibility	Week 1	6.20 ± 0.91 (*n* = 40)	14.0 ± 11.8 (*n* = 40)
	Week 2	6.13 ± 0.91 (*n* = 40)	16.3 ± 12.2 (*n* = 40)
Interrater reproducibility	Researcher 1	6.14 ± 1.04 (*n* = 10)	20.4 ± 12.7 (*n* = 10)
	Researcher 2	5.99 ± 1.10 (*n* = 10)	21.3 ± 13.4 (*n* = 10)

Abbreviations: AIx, augmentation index; PWV, pulse wave velocity.

### 3.1 Pule Wave Velocity

**Figure 1:**
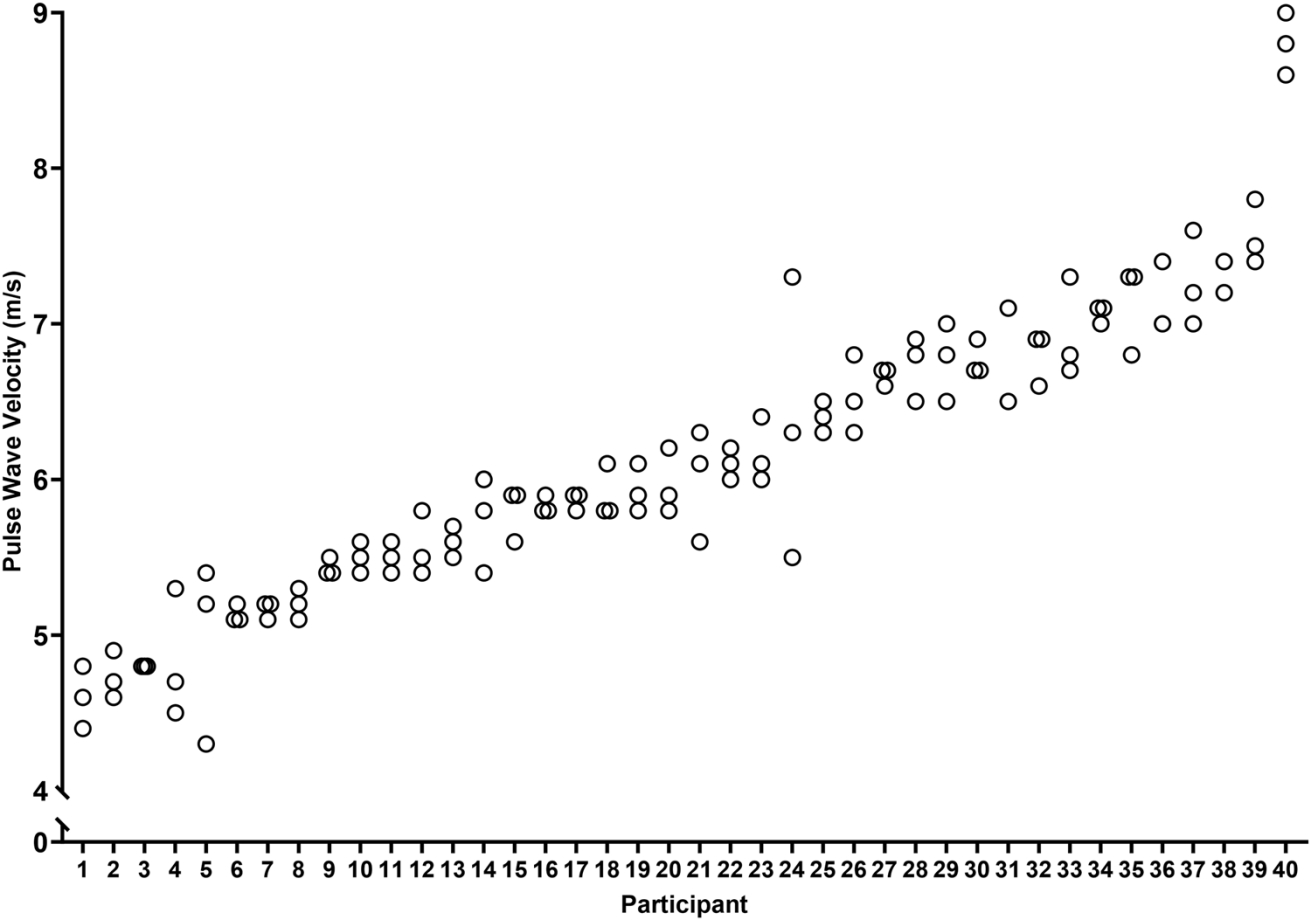
Profile of the triplicate measures obtained by PWV to determine intrarater variation. Participants' individual values were plotted for each repeat in ascending order from the mean.

An excellent ICC was obtained for intratest repeatability (0.92, 95% CI: 0.87–0.95, *p* < 0.05) and interrater reproducibility (0.98, 95% CI: 0.92–1.0, *p* < 0.05). Spread of triplicate results are displayed in Figure 1. There was a good correlation between measurements taken 1 week apart (ICC = 0.87, 95% CI: 0.76–0.93, *p* < 0.05).

### 3.2 Augmentation Index

**Figure 2:**
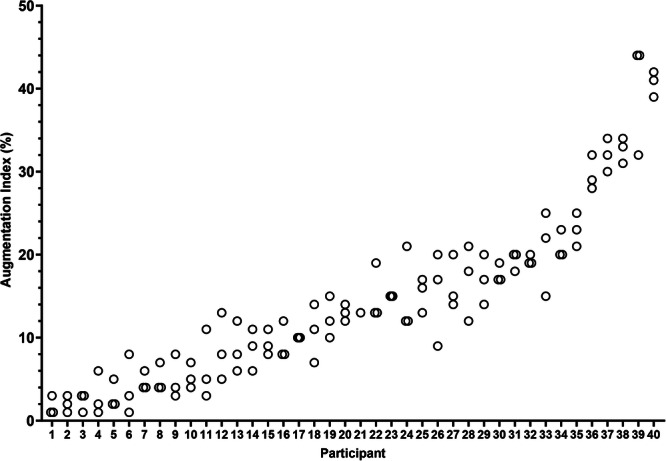
Profile of triplicate measures of augmentation index to determine intratest repeatability. Participants' individual values were plotted for each repeat in ascending order from the mean.

An excellent ICC was obtained for intra‐test repeatability (0.90, 95% CI: 0.84–0.94, *p* < 0.05) and interrater reproducibility (0.93, 95% CI: 0.69–0.982, *p* < 0.05). Triplicate measures are displayed in Figure 2. Measurements taken 1 week apart also showed good correlation (ICC = 0.86, 95% CI: 0.73–0.93, *p* < 0.05).

## 4 Discussion

To the best of our knowledge, this is the first study to assess intra‐test, inter‐rater repeatability, and test‐retest reproducibility of PWV and Alx simultaneously. The primary findings suggest in a sample of healthy adults, there is excellent repeatability and good reproducibility of PWV and Alx measurements.

Intra‐test repeatability has been demonstrated as excellent by Hwang et al. (PWV and Alx [ICC: 0.996 and 0.983, respectively]) with the SphygmoCor Xcel device [23]. This study included both young and older adults (mean: 33.3, range 22–58 years), however due to a small sample size of older adults, it was not possible to determine if repeatability changed between age groups as suggested by Bortel et al. [24].

Making devices less operator dependent is important for use in clinical practice [25]. Novice versus experienced operator analysis has previously been assessed using the SphygmoCor device. Results showed that it takes an estimated 30 participants to ensure acceptable Alx measurements, and around 2.5 h initial training and a further 14 or more practice measures to ensure acceptable PWV measures [24]. The semiautomated device used in this study, requiring far less training, showed excellent reproducibility for both PWV and Alx (ICC: 0.98 and 0.93 respectively).

Day‐to‐day reliability assessed by Hwang et al. showed PWV and Alx reliability to be excellent (ICC = 0.979 and 0.939, respectively) in the Sphygmocor Xcel device [23]. These results indicate higher levels of reproducibility compared to the present study. A difference in study population could be attributed to this finding as the present study only assessed mainly younger participants (range: 22–58 years) and the previous study assessed a larger age range (21–78 years). After 60 years of age, Alx is known to plateau and therefore could yield a more reliable constant measure [26].

Hwang et al. assessed the day‐to‐day and intra‐test reliability of the Xcel and MM3 devices. These devices, both manufactured by SphygmoCor use the semi‐automated (applanation tonometry and oscillometric) and tonometry‐only methods of data acquisition. Measures of PWV and Alx between devices were strongly related (*r* = 0.85 and 0.75), suggesting methods of acquisition are in agreement [27]. Studies in clinical populations found no difference between the Vicorder (using oscillometric technique only) and SphygmoCor SCOR‐Pvx (using gated pulse waveforms obtained through applanation tonometry only) when assessing PWV [28]. Not only did this study evaluate different models of device (each with different algorithms), but there were also different methods of data acquisition (oscillometric vs. applanation tonometry), which gives rise to multiple points of error, as discussed by Bortel et al. [27]. Conversely, Hickison et al. assessed validity and repeatability of the Vicorder apparatus (using oscillometric techniques only) and the SphygmoCor Xcel (semiautomated, carotid application tonometry and femoral cuff) devices. The Vicorder showed a higher degree of accuracy, especially at high PWV, than the semi‐automated SphygmoCor Xcel [29]. This is an important consideration in the trade‐off between being able to assess PWV in the clinic, the assessment being a true representation of a patient's characteristics, and the time taken to obtain the measure (including both clinic time, and the time taken to train the operator).

Intratest repeatability assesses only mechanical and minute‐to‐minute biological variation due to the tape measure distances staying the same between tests. Intrarater repeatability assesses human error as well as mechanical and minute‐to‐minute biological variation. Week‐to‐week reproducibility assesses human error, mechanical variation, day‐to‐day biological variation, and minute‐to‐minute biological variation. With adding additional opportunity for error, we can see that correlation decreases, but it is promising that there was still an excellent level of reproducibility when operators were different.

One of the major limitations of the present study is that it recruited only healthy individuals, and employed only the SphygmoCor Xcel. Healthy participants were recruited to restrict confounding variables, however considering the importance of arterial function evaluation in clinical settings, the present findings should be further confirmed in different clinical populations with gated age groups to allow generalizability of data and further analysis into the variation between devices and methods of acquisition used. Additionally, the SphygmoCor assumes uniform stiffness throughout the artery and also only assesses central artery stiffness. Using ultrasound data acquisition methods may allow researchers/clinicians to pinpoint specific areas of stiffness.

In conclusion, our results show that assessment of PWV and Alx using the SphygmoCor Xcel demonstrate good to excellent repeatability and reproducibility in a sample of healthy adults. Considering high accuracy, noninvasive and easy‐to‐measure features, arterial function measurements may play a vital role and should be integrated in routine clinical practice to facilitate risk stratification in cardiovascular diseases.

## Author Contributions

**Sophie L. Russell:** writing–original draft, investigation, formal analysis, writing–review and editing. **Mushidur Rahman:** investigation, writing–review and editing. **Charles J. Steward:** investigation, writing–review and editing. **Amy E. Harwood:** supervision, writing–review and editing. **Gordon McGregor:** supervision, writing–review and editing. **Prithwish Banerjee:** Supervision, writing–review and editing. **Nduka C. Okwose:** supervision, writing–review and editing, investigation. **Djordje G. Jakovljevic:** conceptualization, writing–review and editing, supervision.

## Conflicts of Interest

The authors declare no conflicts of interest.

## Transparency Statement

The lead author Djordje G. Jakovljevic affirms that this manuscript is an honest, accurate, and transparent account of the study being reported; that no important aspects of the study have been omitted; and that any discrepancies from the study as planned (and, if relevant, registered) have been explained.

## Data Availability

The data that support the findings of this study are available from the corresponding author upon reasonable request.
